# Total Mastectomy Preserving a Ventriculoperitoneal Shunt on the Affected Side: A Case Report

**DOI:** 10.7759/cureus.112483

**Published:** 2026-07-11

**Authors:** Hirofumi Terakawa, Yuki Kurokawa, Ryosuke Mohri, Miki Hirata, Noriyuki Inaki

**Affiliations:** 1 Gastrointestinal Surgery/Breast Surgery, Kanazawa University Hospital, Kanazawa, JPN

**Keywords:** breast cancer, perioperative management, post-mastectomy radiotherapy, radical mastectomy, ventriculoperitoneal (vp) shunt

## Abstract

Ventriculoperitoneal (VP) shunts are the standard treatment for hydrocephalus, and the distal catheter is often routed subcutaneously across the anterior chest wall. The development of ipsilateral breast cancer is rare, with fewer than 15 cases reported in the English literature, and there is no established consensus regarding the management of the shunt catheter during mastectomy.

We report a 58-year-old shunt-dependent woman with a right-sided VP shunt placed for post-subarachnoid hemorrhage hydrocephalus, who was diagnosed with right breast cancer (cT2N1M0, stage IIB). Preoperative imaging demonstrated a 20 mm distance between the medial edge of the tumor and the catheter, which was judged sufficient to allow shunt preservation. A right total mastectomy with axillary lymph node dissection was performed with a neurosurgeon on standby. Energy devices were avoided in the vicinity of the catheter, and sharp dissection was performed with Metzenbaum scissors. The catheter was preserved without damage, and strict aseptic technique was maintained throughout. Adjuvant chemotherapy and post-mastectomy radiotherapy (PMRT) were administered uneventfully after multidisciplinary planning, and the patient remains free from recurrence under ongoing endocrine therapy with abemaciclib.

Total mastectomy with VP shunt preservation can be safely accomplished when a sufficient tumor-catheter margin is confirmed preoperatively and when meticulous intraoperative technique and multidisciplinary collaboration covering PMRT planning are ensured.

## Introduction

Ventriculoperitoneal (VP) shunts are the standard treatment for hydrocephalus, and the distal catheter is often routed subcutaneously or through the breast tissue along the anterior chest wall [1,2]. The occurrence of breast cancer on the same side as a VP shunt is rare; fewer than 15 such cases have been reported in the English-language literature to date [1-6]. Consequently, no consensus exists regarding the management of the shunt catheter during mastectomy.

The clinical dilemma in this setting is whether to preserve the shunt or to reroute or replace it. Each of these options carries specific risks. Intraoperative catheter damage may result in cerebrospinal fluid (CSF) leak and immediate loss of shunt function in a patient who is often shunt-dependent; bacterial contamination of the catheter during mastectomy can ascend to the central nervous system and cause ventriculitis or meningitis, which carry substantial morbidity and mortality; and preservation of subcutaneous tissue around the catheter may, in principle, raise concern for local recurrence if oncological clearance is compromised. These considerations explain why the literature has previously favored shunt rerouting whenever the tumor lies close to the catheter and why a clear preoperative framework, including alternative neurosurgical strategies if intraoperative damage occurs, is essential. Herein, we report a case of right breast cancer ipsilateral to a VP shunt in which preoperative imaging confirmed a safe distance between the tumor and the catheter, allowing a total mastectomy with successful shunt preservation, and we discuss the perioperative decision pathway together with a comparison to previously reported cases.

## Case presentation

A 58-year-old woman was referred to our department for a right breast mass detected on screening ultrasound. Seven years earlier, she had suffered a subarachnoid hemorrhage due to a ruptured cerebral aneurysm, complicated by communicating hydrocephalus, for which a right-sided programmable-valve VP shunt (Codman Hakim programmable valve, opening pressure setting 120 mmH2O) had been placed. The distal catheter was routed through the right side of the neck and along the right sternal border. There had been no shunt revision since the initial placement, and the patient was clinically shunt-dependent: prior attempts at gradual valve uptitration had been associated with symptomatic intracranial hypertension, and the neurosurgical team confirmed that shunt removal was not a feasible option for this patient.

Screening background and detection

In Japan, the standardized pre-screening questionnaire administered before mammography routinely includes explicit items asking whether the examinee has an indwelling VP shunt or a central venous (CV) port. In customary clinical practice, mammography is generally not performed in women with an indwelling VP shunt, and this convention is widely shared by both clinicians and patients. Consistent with this practice, the present patient, who had been shunt-dependent since the treatment of her ruptured cerebral aneurysm, had understood for many years that she "could no longer undergo mammography". As a consequence, her breast cancer screening had lapsed for several years after shunt placement. Her current breast lesion was eventually detected incidentally when she happened to undergo an opportunistic breast ultrasound examination, which prompted referral to our institution. We respected her established preference and continued to use ultrasound and magnetic resonance imaging (MRI) rather than mammography for diagnostic workup.

**Figure 1:**
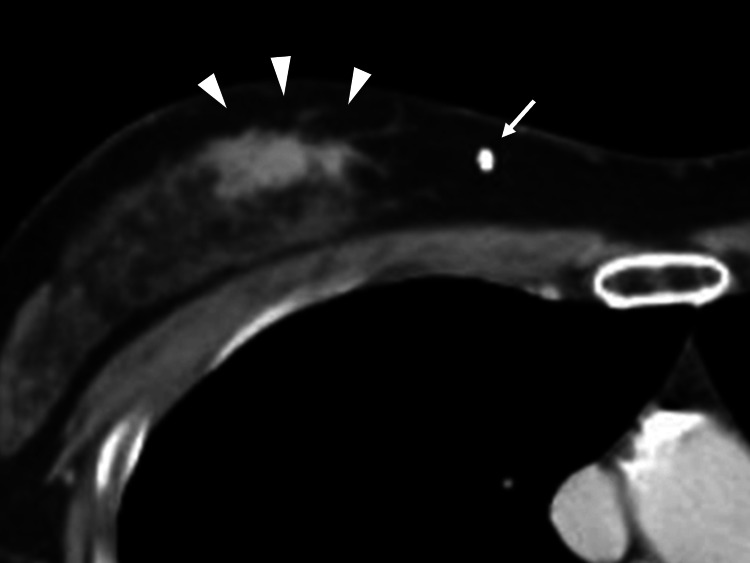
Contrast-enhanced computed tomography (axial section): preoperative tumor-catheter relationship An irregularly shaped, enhancing tumor is seen in the right breast (arrowheads). The shunt catheter is identified as a high-density structure on the medial side of the tumor (arrow).

**Figure 2:**
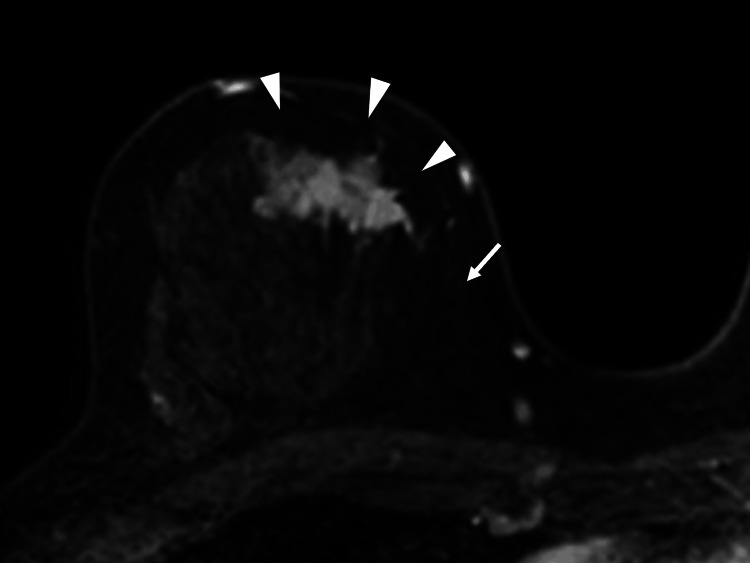
Contrast-enhanced magnetic resonance imaging (axial section): tumor-catheter distance measurement Similar to the computed tomography findings, an irregularly enhancing mass is seen in the right breast (arrowheads), with the shunt catheter located on the medial side (arrow). The medial edge of the tumor is approximately 20 mm from the catheter.

On physical examination, a palpable mass was present in the right breast. Breast ultrasonography showed a 35 mm irregular hypoechoic mass in the upper inner quadrant. Contrast-enhanced computed tomography (CT) demonstrated an enhancing tumor in the right breast and right axillary lymph node swelling, without distant metastasis (Figure 1). Contrast-enhanced MRI was performed to evaluate the precise extent of the tumor and the tumor-catheter distance (Figure 2); the medial edge of the tumor was approximately 20 mm from the shunt catheter. To ensure shunt valve safety, skull radiography was performed before and after MRI to verify the pressure setting and stability of the valve, and no change was observed. Core needle biopsy of the breast mass yielded a diagnosis of invasive ductal carcinoma, and fine-needle aspiration cytology of the axillary lymph node was positive for metastasis. The clinical diagnosis was cT2N1M0, stage IIB. Immunohistochemical profiles of the biopsy specimen and of the final mastectomy specimen are summarized in Table 1.

**Table 1:** Comparison of immunohistochemistry on the preoperative core needle biopsy and the final mastectomy specimen

Marker	Core needle biopsy (preoperative)	Mastectomy specimen (final)
Estrogen receptor (ER)	Positive, 95%	Positive, 95%
Progesterone receptor (PR)	Positive, 80%	Positive, 60%
HER2 (immunohistochemistry score)	0 (negative)	0 (negative)
Ki-67 labeling index	12.9%	16%

Preoperative multidisciplinary planning and intraoperative contingency

Given the 20 mm distance between the tumor and the shunt catheter on preoperative imaging, we judged that the catheter could be safely preserved. The procedure was planned jointly with the neurosurgery team, and a defined contingency pathway was established in case of intraoperative catheter damage: First, although the neurosurgical team was not physically stationed inside the operating theatre during the mastectomy, the team remained on standby for immediate consultation and intervention. A clear intraoperative communication pathway was established in advance, such that the breast surgical team would contact the on-call neurosurgeon without delay if any injury to the distal catheter were suspected. Second is the primary repair or intraoperative replacement of the distal catheter with contralateral rerouting in the event of catheter laceration. Third is the complete removal of the shunt system with temporary external ventricular drainage and delayed reconstruction in the event of severe contamination or irreparable injury. Lastly, endoscopic third ventriculostomy (ETV), although capable of eliminating the need for hardware in selected patients, was considered inappropriate here because the patient had post-hemorrhagic communicating hydrocephalus and confirmed shunt dependency, settings in which ETV is known to be markedly less successful than in obstructive hydrocephalus.

Operation

**Figure 3:**
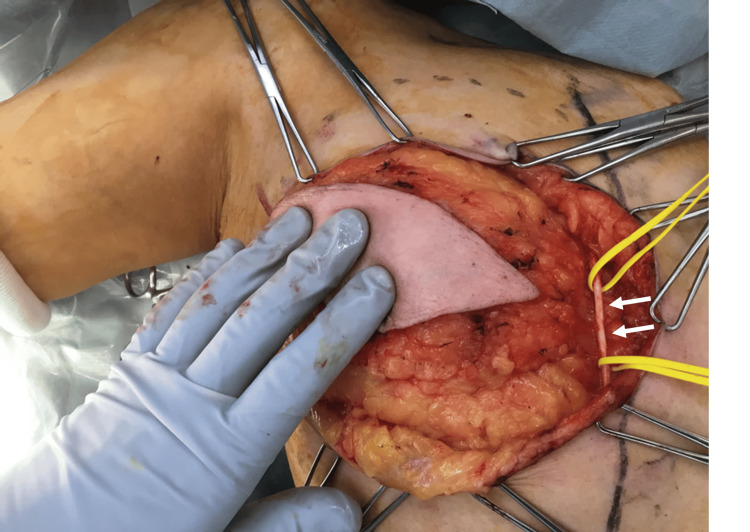
Intraoperative photograph before tumor removal, after the elevation of the skin flap The shunt catheter (arrows) is identified within the subcutaneous tissue and isolated using vessel loops; sharp dissection was performed with Metzenbaum scissors to avoid thermal injury. The breast and tumor are still in situ.

**Figure 4:**
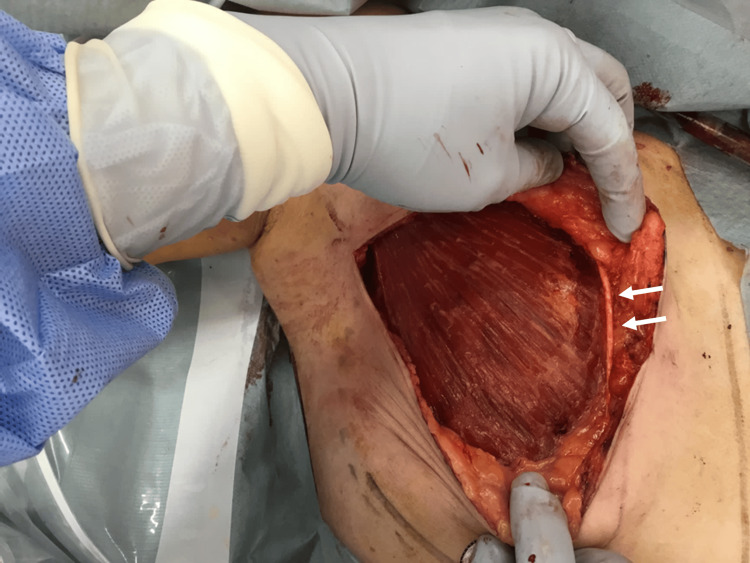
Intraoperative photograph immediately after total mastectomy The catheter (arrows) is completely exposed and preserved on the pectoralis major fascia, without any damage to the catheter, after the breast and tumor have been removed.

The patient underwent right total mastectomy and axillary lymph node dissection. During elevation of the skin flap, the course of the shunt catheter was identified. To prevent thermal injury, we avoided the use of energy devices in the vicinity of the catheter and performed careful sharp dissection with Metzenbaum scissors (Figure 3, intraoperative photograph showing the catheter identified within the subcutaneous tissue and isolated with vessel loops before tumor removal). The catheter was completely exposed and preserved within the subcutaneous tissue on the pectoralis major fascia (Figure 4, intraoperative photograph after total mastectomy showing the fully exposed and undamaged catheter on the pectoralis major fascia). For infection control, the surgical field was thoroughly irrigated, and surgical gloves were exchanged before wound closure. The operative time was 154 minutes, and the estimated blood loss was 43 mL. The postoperative course was uneventful, with no signs of surgical site infection or shunt malfunction.

Final pathology and adjuvant therapy

Histopathological examination revealed invasive ductal carcinoma with an invasive component measuring 34 mm in greatest dimension. Lymph node metastases were identified in four of the dissected axillary nodes, yielding a final pathological diagnosis of pT2N2M0, stage IIIA. The immunohistochemical profile is shown in Table 1. Adjuvant chemotherapy consisting of dose-dense epirubicin and cyclophosphamide followed by dose-dense paclitaxel was administered. Post-mastectomy radiotherapy (PMRT) was subsequently performed after multidisciplinary discussion with the neurosurgery and radiation oncology teams, who confirmed that the planned dose and field would not adversely affect the catheter material or shunt function. During PMRT, particular attention was paid to the relationship between the irradiation field and the subcutaneous catheter. The daily skin condition over the catheter and along the wound was monitored, and no acute radiation dermatitis severe enough to threaten tube exposure was observed. At present, the patient is receiving endocrine therapy combined with abemaciclib and remains free from recurrence.

## Discussion

The management of a VP shunt during breast cancer surgery is a clinically important but uncommon issue, and to date, fewer than 15 cases have been reported in the English-language literature [1-6]. A comparison of representative published cases and the present case is shown in Table 2.

**Table 2:** Comparison of the present case with previously reported cases of breast cancer ipsilateral to a VP shunt PMRT: post-mastectomy radiotherapy; VP: ventriculoperitoneal

Author (year)	Tumor-catheter relationship	Shunt management	Adjuvant radiotherapy	Outcome
Keshtgar et al. (2001) [1]	Catheter distant from the tumor	Preserved	Not specified	Uneventful
Lee et al. (2010) [4]	Multi-centric tumor, catheter distant	Preserved	Performed	Uneventful
Jain and Kokan (2013) [3]	Tumor encasing the catheter	Revised (rerouted)	Performed	Uneventful
Roka et al. (2010) [6]	Tumor compressing the distal catheter	Shunt failure due to compression	-	Required revision
Copeland-Halperin and Cohen (2015) [5]	Recurrence near the preserved catheter	Previously preserved	Previously performed	Recurrence reported
Present case (2025)	20 mm margin between the tumor and catheter	Preserved	Performed (PMRT)	No recurrence

Previous reports suggest that shunt rerouting is generally preferred when the tumor is in close contact with the catheter or invades the surrounding tissue, as illustrated by Jain and Kokan, in whom the tumor encased the catheter and necessitated shunt revision [3]. In contrast, preservation of the shunt has been considered feasible when a sufficient safety margin exists between the tumor and the catheter [1,4]. In our case, a 20 mm margin allowed us to perform the mastectomy safely without compromising the shunt. Nevertheless, careful follow-up is warranted, as both local recurrence in the preserved breast tissue [5] and shunt malfunction due to tumor compression [6] have been described. Preoperative evaluation with CT and MRI is therefore essential to define the precise spatial relationship between the tumor and the catheter and to allow a contingency plan to be agreed upon with neurosurgery before incision.

Nodal upstaging and oncological implications

In the present case, there was a notable discrepancy between clinical and pathological nodal status (cN1 → pN2, with four positive axillary nodes). This kind of upstaging is clinically relevant when shunt preservation is being considered, because a higher than expected nodal burden may modestly increase the local recurrence risk and intensifies the requirement for PMRT, which in turn brings the irradiation field closer to the preserved catheter. We therefore consider that the decision to preserve a shunt should not be made on imaging alone, but should be revisited intra- and postoperatively in light of the final pathological staging. In our case, because final staging remained locoregional (stage IIIA), and because multidisciplinary PMRT planning was able to spare the catheter, we judged that shunt preservation remained appropriate.

Screening considerations

The optimal modality for breast cancer screening in women with a VP shunt remains poorly defined. Although there is no robust published evidence that standard mammographic compression directly damages a VP shunt or its distal catheter, avoidance of mammography in shunt-bearing patients has become an established custom in Japanese clinical practice. The national pre-screening questionnaire for mammographic screening explicitly inquires about the presence of a VP shunt or a CV port, and in routine practice, such patients are typically excluded from compression mammography. As illustrated by the present case, this customary avoidance, combined with patients' own conviction that mammography is no longer feasible after shunt placement, can result in a multi-year lapse in screening and may therefore contribute to delayed detection of breast cancer in this population. Active promotion of breast ultrasound, supplemented by MRI when clinically indicated, may provide a pragmatic and patient-acceptable alternative that facilitates earlier detection while accommodating both patient anxiety and prevailing clinical custom. Further studies are warranted to establish evidence-based screening recommendations specifically for women with a VP shunt.

Infection prevention

With regard to surgical technique, prevention of infection is paramount because shunt infection may result in meningitis. We adhered strictly to aseptic technique, including extensive irrigation and glove exchange before wound closure, in accordance with the Centers for Disease Control and Prevention guidelines for the prevention of surgical site infection [7].

PMRT

PMRT also deserves specific consideration in patients with a VP shunt. Previously reported risk factors that increase the likelihood of radiation-related complications in this setting include proximity of the postoperative wound to the shunt, superficial location of the catheter, thin or scarred overlying skin, the need for bolus application, and impaired wound healing due to diabetes mellitus, steroid use, or prior irradiation [1,2,4]. Under such conditions, radiation-induced skin toxicity within the treatment field may lead to tube exposure, secondary shunt infection, and delayed wound healing. In the present case, the catheter had been exposed intraoperatively and was located within the wound-healing area, and Figures 1-2 indicate that it was relatively superficial; these factors were considered to increase the theoretical risk of shunt-related complications during PMRT. Conversely, current clinical evidence does not suggest that therapeutic doses of external beam radiotherapy directly cause immediate material degradation or rupture of the catheter, and several reports have described uneventful PMRT in patients with an in situ shunt catheter [1,2,4]. Taking these considerations together, PMRT in our patient was delivered after multidisciplinary planning with the neurosurgery and radiation oncology teams to optimize the field arrangement and dose distribution relative to the catheter, with close monitoring of the overlying skin and wound throughout the treatment course. No radiotherapy-related shunt complication was observed. 

## Conclusions

We successfully treated a patient with breast cancer ipsilateral to a VP shunt by total mastectomy with shunt preservation, followed by uneventful PMRT. Careful preoperative assessment of the tumor-catheter relationship, a prespecified intraoperative contingency plan agreed with neurosurgery, meticulous surgical technique avoiding thermal and mechanical injury, strict aseptic technique to prevent meningitis, and multidisciplinary planning of PMRT are essential to safely combine oncological and neurosurgical objectives in this rare clinical setting.
